# Addressing alcohol dependence in primary care: longitudinal registry-based study of practitioner activity following new policy and access to training

**DOI:** 10.1093/eurpub/ckaf060

**Published:** 2025-06-10

**Authors:** Karin Hyland, Anders Hammarberg, Erik Hedman-Lagerlöf, Olle Wiklund, Ingvar Rosendahl, Sven Andreasson, Per Nilsen

**Affiliations:** Department of Clinical Neuroscience, Karolinska Institutet, Stockholm, Sweden; Centre for Psychiatry Research, Department of Clinical Neuroscience, Karolinska Institutet & Stockholm Health Care Services, Stockholm, Sweden; Stockholm Centre for Dependency Disorders, Stockholm Health Care Services, Stockholm, Sweden; Centre for Psychiatry Research, Department of Clinical Neuroscience, Karolinska Institutet & Stockholm Health Care Services, Stockholm, Sweden; Stockholm Centre for Dependency Disorders, Stockholm Health Care Services, Stockholm, Sweden; Division of Psychology, Department of Clinical Neuroscience, Karolinska Institutet, Stockholm, Sweden; Gustavsberg University Primary Care Center, Stockholm Health Care Services, Stockholm, Sweden; Centre for Psychiatry Research, Department of Clinical Neuroscience, Karolinska Institutet & Stockholm Health Care Services, Stockholm, Sweden; Centre for Psychiatry Research, Department of Clinical Neuroscience, Karolinska Institutet & Stockholm Health Care Services, Stockholm, Sweden; Centre for Psychiatry Research, Department of Clinical Neuroscience, Karolinska Institutet & Stockholm Health Care Services, Stockholm, Sweden; Stockholm Centre for Dependency Disorders, Stockholm Health Care Services, Stockholm, Sweden; Department of Global Public Health, Karolinska Institutet, Stockholm, Sweden; Department of Health, Medicine and Caring Sciences, Linköping University, Linkoping, Sweden; School of Health and Welfare, Halmstad University, Halmstad, Sweden

## Abstract

The present study aimed to investigate the extent to which two new implementation strategies—a new policy mandating alcohol interventions in primary care and access to online training, impacted alcohol-related clinical activities in primary care in Stockholm. This was a prospective longitudinal register-based study. One hundred twenty-nine primary care clinics in Region Stockholm agreed to provide data. The new healthcare policy was introduced in February 2021. A brief digital training for primary care professionals on managing harmful alcohol use and dependence was launched 10 months later. Seven indicators that reflect alcohol-related clinical activities were obtained from electronic case files: structured documentation on alcohol habits, the AUDIT instrument, ordering of blood tests for biomarkers of heavy drinking, prescription of medicines for alcohol dependence, registered alcohol-related diagnoses, completed advice regarding alcohol use disorder (AUD), and referrals to specialized care. Data from registers were collected before and after the policy and training was available. At baseline low levels of alcohol-related clinical activities were found in primary care. A modest, clinically non-significant increase was seen for all indicators except for frequency of prescription of medicines for alcohol dependence, over the whole follow-up. The digital training was not associated with an increase in alcohol-related clinical activities. While a policy making alcohol interventions mandatory, combined with a training program, has strong support from implementation science, only a modest, clinically non-significant increase in alcohol-related clinical activities was found. Stronger implementation strategies seem necessary to improve management of alcohol dependence in primary care.

## Introduction

Recent research has found that people with alcohol dependence of moderate severity can be helped with relatively limited treatment within primary care [1]. Thus, primary care provides an opportune setting both for addressing heavy drinking as a risk factor for disease and for treating alcohol dependence. However, while primary care in many countries is mandated to treat harmful use of alcohol and dependence, it is unclear to what extent this policy has been translated into clinical practice [2–6].

Healthcare in Sweden is organized at the regional level, with each of the country’s 21 regions responsible for providing healthcare services to its residents. The regions are responsible for managing and funding healthcare services, including hospitals, primary care clinics, and specialized healthcare services. The healthcare system is funded through taxes and is primarily publicly funded, with private healthcare providers also playing a role. Patients have the freedom to choose their healthcare provider and have access to a wide range of services, including preventive care, medical treatment, and rehabilitation services.

In this paper, we aimed to chart and analyse the level of alcohol-related clinical activities in Region Stockholm following the introduction of a new policy, making alcohol interventions mandatory in primary care. The new policy which came into force in Region Stockholm in 2021, coincided with the launch of a web-based training program focused on managing alcohol use disorders using the 15-Method [7]. This training opportunity was made available to all clinicians in the region.

The 15-Method has been proposed as a feasible method to address and to treat harmful use and dependence of alcohol in primary care settings [7]. The method has a three-step approach, where the first step is pragmatic, or targeted, screening [8]. The second step involves a more thorough assessment, based on ‘The Drinker’s Check-Up’ [9]. The third step involves pharmacotherapy and/or brief psychotherapy. A large randomized controlled trial (RCT) found that treatment for alcohol dependence in primary care with the 15-Method, based on a one-day training for physicians, was equally efficacious in reducing alcohol consumption as treatment in specialized care [1]. The name of the method refers both to the length of the sessions, 15 min, and that the target group for the intervention in the second and third steps are patients who score 15 points or above on the Alcohol Use Disorders Identification Test (AUDIT) [10]. The AUDIT is a screening tool that consists of 10 questions that evaluate alcohol intake, dependence symptoms, and alcohol-related harm.

While research indicates that training alone is insufficient to achieve increased alcohol activity in primary care settings, it is clear that successful alcohol interventions require support from upper-level management and adequate resources [11–15]. With the implementation of the new policy and the availability of online training in the 15-Method in Region Stockholm, Sweden, we were able to conduct a longitudinal registry-based study to examine the impact on alcohol-related clinical activities. Our aim was to investigate clinicians’ identification, assessment, and treatment of harmful use and alcohol dependence in primary care and examine if these indicators changed over measurement occasions as a function of policy shift and availability to online training in the 15-Method.

## Methods

### Study design

This is a prospective longitudinal study within primary care in Region Stockholm, building on data extracted from primary care electronic case files at six measurement occasions: three months before the new policy was introduced; 3 and 9 months after the new policy came into force, but before training in the 15-Method was made available; and 6, 12, and 18 months after training was made available. Analyses included the whole population of clinicians in the participating primary care clinics, investigating if changes in their alcohol-related clinical activities coincided with the introduction of the policy and availability of online training. We did not specifically follow-up the group of clinicians who took part in the online training.

### Participants and setting

In this study, 129 primary care clinics, representing 58% of the 223 clinics in Region Stockholm, were approached, agreed to provide data, and remained active throughout the entire study period. Region Stockholm is the authority responsible for publicly funded healthcare provision to Stockholm’s approximately 2.4 million inhabitants. As an expression of the stakeholder desire to bring about a change in the management of harmful alcohol use and dependence, all primary care clinics are since 1 February 2021, required to manage patients who do not require the expertise of specialized care. This shift in policy was not coupled with changes in economic reimbursements or systematic follow-up of the primary care clinics’ work related to alcohol dependence management. However, since late November 2021, a web-based training program was made freely available for all primary care clinicians (see below).

### Web-based training in the 15-Method

Following the introduction of the new policy, brief online training in the management of harmful use of alcohol and dependence was offered by Region Stockholm to all professions at the primary care clinics in Region Stockholm. The main learning objective was to support primary care clinics to practice the 15-Method in their daily work.

### Procedure and indicators

The main level of analysis was the primary care clinic and applied to all alcohol-related clinical activities performed by all professions. Data on quantity of each indicator on alcohol-related clinical activities were collected at six measurement occasions. Each occasion lasted one month. In this way, each primary care clinic constituted its own control.

Data were obtained from the primary care electronic case files with the goal of including clinical activities targeted at each of the three steps in the 15-Method: (1) raising the topic of alcohol, (2) assessing the severity of the disorder, and (3) pharmacological and psychological treatment of alcohol dependence. In this registry-based study, we found seven indicators in the electronic case files reflecting primary care clinicians’ real-world alcohol-related activities. These were followed over measurement occasions, making this a mirror-image study, with a baseline prior to the policy shift and measurements at a number of follow-up points. The choice of seven indicators was dictated by the information available in the electronic case files. The specific indicators were:

- Raising the topic about alcohol consumption: This was measured by the frequency of
  (1) Structured documentation on alcohol habits: weekly alcohol consumption and heavy drinking occasions.
- Assessing the severity of the disorder:
  (2) Use of the Alcohol Use Disorders Identification Test (AUDIT) [10].
  (3) Ordering of blood tests for biomarkers of heavy drinking, carbohydrate deficient transferrin (CDT) or phosphatidylethanol (PEth).
- Treatment of alcohol dependence:
  (4) Prescription of medicines for alcohol dependence (naltrexone, nalmefene, acamprosate, disulfiram).
  (5) Registered alcohol-related diagnoses: harmful use of alcohol F101, alcohol dependence, F 102.
  (6) Completed brief advice and extended advice regarding hazardous use, harmful use, and dependence of alcohol, registered using KVÅ-codes, Klassifikation av Vårdåtgärder (Classification of care measures).
  (7) Referrals to specialized addiction care.

### Data analysis

The frequency of each of the seven indicators was divided by the number of patients who were listed at each primary care clinic at each study period. The quota was then multiplied by 10 000 so the outcome in the analyses becomes the incidence per 10 000 patient-months. A Care Need Index (CNI) was included in all the models to the seven indicators. This index is provided by Statistics Sweden and uses socioeconomic conditions to identify risk for illness in the area where the primary care clinic is located [16]. An index value higher than one indicates a higher need of care than the average in the region and an index value lower than one indicates a lower need of care than the average.

The CNI variable was centred which is a useful tool for interpretation when predictors do not have a meaningful zero point. Centring predictor variables becomes even more important when dealing with interaction terms and therefore especially in multilevel models where cross-level interactions are very common. Besides, that centring makes the model more interpretable, the calculations tend to go faster and encounter fewer convergence problems. Grand mean centring is most often used, but it is also possible to centre on a theoretically interesting value [17]. In this study, the CNI values were centred around the value of one which results in a centred variable where a value of zero indicates a primary care clinic with the same risk for illness as Region Stockholm in general. A positive value indicates a primary care clinic with higher risk for illness and consequently, a negative value indicates a primary care clinic with lower risk for illness compared to Region Stockholm in general. The measurement occasions will usually be thought of as going from 1 to 6 in a study with six occasions like this study. However, for the same reason as the main reason for centring variables, which is to get an interpretable intercept, the measurement occasions *T* are coded as *t* = 0, 1, 2, 3, 4, 5.

Multilevel linear models, or more precisely Growth curves models, were used for the analyses of the seven indicators measured at six measurement occasions. Models with increasing complexity were compared against each other with the likelihood-ratio test (LRT), to find the best model fit, i.e. random intercept, random slope, modelling the covariance structure, interactions, and adding higher-order polynomials. The presence of higher-order polynomials would indicate ‘jumps’ in the data which therefore consequently do not follow a linear trend, possibly as an effect of an event that occurred during measurement occasions. In the presence of higher-order polynomials in a model, data will thereafter be analysed with piecewise growth curve regression as a final model. In the absence of higher-order polynomial, estimates for the best model fit for each indicator will be presented.

The fixed part in multilevel regression resembles ordinary single-level regression when it comes to interpreting the regression coefficients. For the random part of the models, the standard deviation of the intercept (baseline) and the slope (occasion) tell us how much the primary care clinics vary at baseline and how much they vary in their development during measurement occasions. If these standard deviations are statistically significant, the need for random models is justified. The correlation between intercept and occasion is not only interesting in how strong the correlation is but also the sign of the value is interesting. If the sign is negative, it means that a higher value in anyone of the intercept or occasion leads to a lower value in the other, while a positive sign means that a higher value in one of them also leads to a higher value in the other.

The study investigates behaviour change among all primary care clinicians at participating primary care clinics in Region Stockholm. Power calculation was not needed since the study concerned an entire population. All analyses were performed using R Statistical Software [18] and the R Package NLME: Linear and Nonlinear Mixed Effects Models [19].

## Results

The raw actual frequency, displayed as the incidence per 10 000 patient-months of the seven reported indicators, is presented for each measurement occasion in Table 1. Overall, low levels of activity were found. There was a slight increasing trend during measurement occasions but without any dramatic increase after the new policy was introduced, which is marked in Table 1 as a dashed horizontal line between baseline and three months post policy. The time point when the training in the 15-Method was made available is marked with the second dashed horizontal line between nine months post policy and six months after training was made available.

**Table 1.** The incidence per 10 000 patient-months of seven alcohol-related indicators during measurements occasions for 129 participating PC-clinics

Occasion	Incidence per 10 000 patient-months of:						
	1. Structured documentation on alcohol habits	2. Use of the AUDIT instrument	3. Ordering of blood tests for biomarkers of heavy drinking	4. Prescription of medicines for alcohol dependence	5. Registered alcohol-related diagnoses	6. Completed advice regarding Alcohol use disorder	7. Referrals to specialized addiction care
0. Baseline	66.01	3.80	3.59	0.83	3.71	4.57	0.71
1. Three months post policy	60.91	3.70	3.72	0.76	4.26	3.98	0.65
2. Nine months post policy	73.09	4.65	5.55	1.04	4.79	4.42	0.79
3. Six months after training was made available	80.72	4.31	5.57	0.80	5.04	4.06	0.68
4. 12 months after training was made available	84.16	6.42	6.82	0.82	5.95	5.13	1.03
5. 18 months after training was made available	87.53	4.99	7.05	1.02	5.57	4.07	0.98

**Figure 1.**
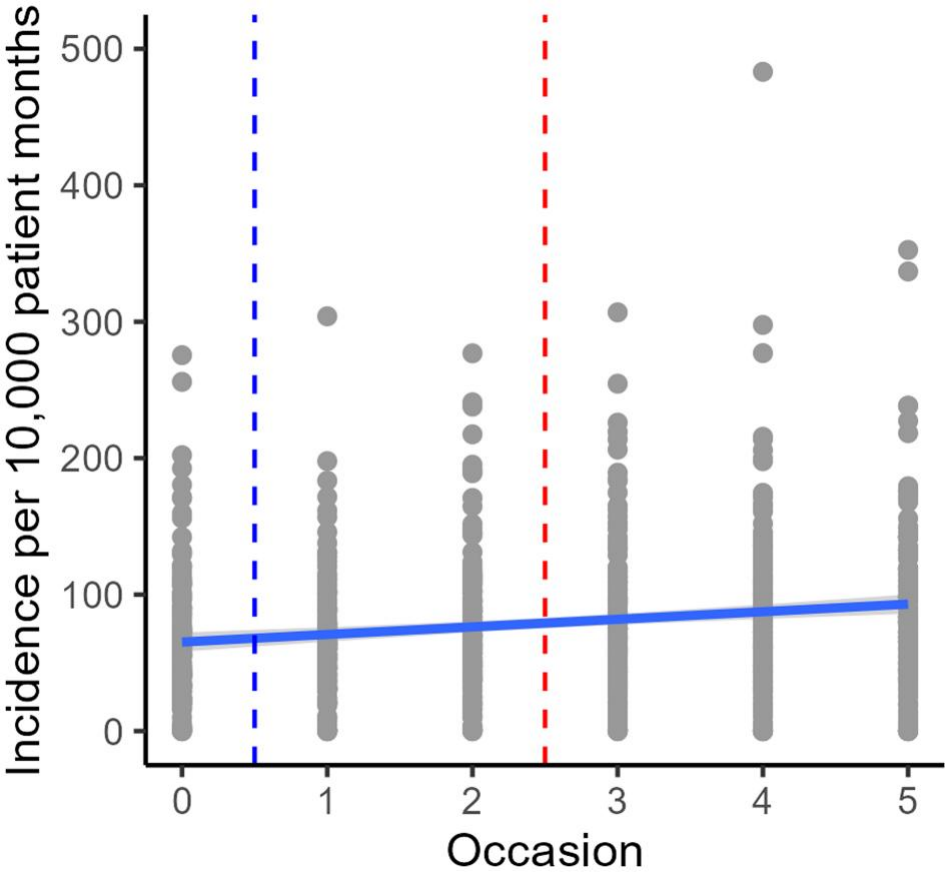
Trajectory for structured documentation on alcohol habits over measurement occasions.

Structured documentation on alcohol habits was the most frequently registered indicator. Since the frequency was so much higher for structured documentation compared to the other indicators, the trend over measurement occasions for structured documentation is displayed in Fig. 1 while the trend for the remaining six indicators are displayed in Supplementary Fig. S1 and together confirms the impression from Table 1. Be aware that the scale of the *y*-axis is different in Fig. 1 and Supplementary Fig. S1.

The fixed effects of the growth curve models are presented in Table 2. As shown in Table 2, there was a positive increased linear trend during the measurement occasions for all indicators except for one, which was prescription of medicines for alcohol dependence. Although, the trends for six of the indicators were statistically significant, they are hardly clinically significant. For instance, structured documentation on alcohol habits was found 4.6 times more often per 10 000 patient-months. Structured documentation on alcohol habits was also the only indicator with a statistically significant interaction between occasion and CNI which means that the increasing trend during measurement occasions for this indicator was not independent of CNI. Instead, primary care clinics with higher expected rates of illness also had a higher increasing trend of structured documentation on alcohol habits compared to primary clinics with lower expected rates of illness. For two indicators, use of the AUDIT instrument and prescription of medicines for alcohol dependence, the CNI was statistically significant but only at baseline and in a negative direction.

**Table 2.** Growth curve models for seven alcohol-related indicators—fixed effects

	*β*	SE *β*	95% CI
Frequency of structured documentation on alcohol habits			
Intercept	62.338	4.052	54.402, 70.275
Occasion	4.606	1.009	2.630, 6.583
CNI	19.841	8.756	2.557, 37.124
Occasion × CNI	6.489	2.180	2.219, 10.760
Frequency of use of the AUDIT instrument			
Intercept	3.961	0.503	2.976, 4.946
Occasion	0.313	0.118	0.081, 0.545
CNI	−2.336	0.958	−4.228, −0.445
Frequency of ordering of blood tests for biomarkers of heavy drinking			
Intercept	3.203	0.280	2.654, 3.752
Waves	0.571	0.103	0.368, 0.774
Frequency of prescription of medicines for alcohol dependence			
Intercept	0.948	0.066	0.818, 1.078
CNI	−0.560	0.143	−0.843, −0.276
Frequency of registered alcohol-related diagnoses			
Intercept	3.643	0.223	3.207, 4.080
Occasion	0.410	0.062	0.288, 0.532
Frequency of completed advice regarding alcohol use disorder			
Intercept	3.651	0.225	3.209, 4.092
Occasion	0.403	0.061	0.283, 0.522
Frequency of referrals to specialized addiction care			
Intercept	0.645	0.071	0.505, 0.785
Occasion	0.055	0.023	0.010, 0.099

Note: Intercept expresses the incidence per 10 000 patient-months for the indicator at baseline. Occasion expresses the mean change in the indicator per 10 000 patient-months during measurement occasions.

Another noteworthy result to notice in Table 2 is that no higher-order polynomials are present for any of the seven indicators, which indicates that neither the new policy nor the training in the 15-Method had any noticeable effect on the increasing trend.

In Table 3, the random effects of the growth curve models are displayed. A random intercept and slope model were justified for five of the indicators while only a random intercept model was needed for prescription of medicines for alcohol dependence, and for referrals to specialized addiction care. However, there is a distinction between the two indicators. For the prescription of medicines for alcohol dependence there was no need for the occasion variable (the slope) in the model which means that the value at baseline (the intercept) is valid during measurement occasions and that no change is present. For the indicator referrals to specialized addition care, the addition of random slope to the model had a very small impact on model fit and was omitted from the final model. The negative sign of the correlation between intercept and occasion in the model for structured documentation on alcohol habits means that a higher frequency of documentation at baseline gives a lower increase during measurement occasions or *vice versa*.

**Table 3.** Growth curve models for seven alcohol-related indicators—random effects

	Estimates	95% CI
Frequency of structured documentation on alcohol habits		
SD (intercept)	39.002	33.613, 45.256
SD (occasion)	8.795	7.324, 10.561
Cor (intercept, occasion)	−0.036	−0.214, 0.144
Frequency of use of the AUDIT instrument		
SD (intercept)	3.848	2.941, 5.035
SD (occasion)	0.620	0.300, 1.279
Cor (intercept, occasion)	−0.033	−0.423, 0.368
Frequency of ordering of blood tests for biomarkers of heavy drinking		
SD (intercept)	2.261	1.766, 3.894
SD (occasion)	0.911	0.739, 1.121
Cor (intercept, occasion)	0.530	0.034, 0.816
Frequency of prescription of medicines for alcohol dependence		
SD (intercept)	0.567	0.466, 0.689
Frequency of registered alcohol-related diagnoses		
SD (intercept)	1.867	1.486, 2.346
SD (occasion)	0.428	0.302, 0.607
Cor (intercept, occasion)	0.194	−0.304, 0.609
Frequency of completed advice regarding alcohol use disorder		
SD (intercept)	1.613	0.783, 3.323
SD (occasion)	0.264	0.016, 4.358
Cor (intercept, occasion)	0.918	−1.000, 1.000
Frequency of referrals to specialized addiction care		
SD (intercept)	0.220	0.115, 0.420

## Discussion

We found a small positive increase in six of seven alcohol-related clinical activities following the introduction of the new policy that remained the whole follow-up period. However, the availability of the web-based training in the 15-Method was not associated with an additional increase in alcohol-related clinical activities.

Before the implementation of the new policy, minimal alcohol-related activities were documented in primary care in Region Stockholm. Our findings are consistent with data from the National Board of Health and Welfare, where repeated surveys since 2014 have shown that less than 1% of all patients in Sweden are offered interventions for unhealthy drinking habits. No progress was made in addressing this issue between 2013 and 2021 period [20].

Throughout the two-year follow-up period of this study, a positive linear trend was observed for all indicators except prescription of medicines for alcohol dependence. It is important to note that the increases were minimal, as they occurred from initially low levels. Surveys conducted in Stockholm and its surrounding municipalities show that 18% of men and 15% of women have hazardous alcohol consumption [21], exceeding the recommended limits of 14 standard drinks per week for men and nine for women, or more than five drinks on a single occasion once per month or more often for men and four for women (the Swedish hazardous alcohol consumption levels at the time of the study). These findings highlight a significant contrast between the prevalence of hazardous alcohol consumption and the low levels of alcohol-related clinical activities documented in our registry-based study, suggesting a gap between the need for care and its accessibility.

The new policy on alcohol interventions in primary care in Region Stockholm was a concerted effort to develop a new strategy to improve the prevention and treatment of harmful use and alcohol dependence. Most studies that have investigated implementation of alcohol interventions in primary care and other settings have focused on one supportive strategy at a time, e.g. financial incentive or training of healthcare professionals. However, research in implementation science shows that successful implementation usually requires the use of more than one strategy because there is typically a need to address numerous barriers to implementation of interventions [22, 23]. Furthermore, alcohol intervention implementation projects that have used a combination of financial incentives and training programs have mostly been time-limited projects, typically with an emphasis on training. Strong professional support for a clinical area, good infrastructural support, evidence-based interventions and screening-tools that are not too time-consuming have been found to facilitate implementation [12]. A review by Kaner [11] concluded that brief alcohol interventions cannot occur without some form of senior management support. However, management strategies to support implementation have usually not been included in projects to promote alcohol intervention.

Improving the implementation of effective interventions remains a major challenge in preventing and treating alcohol-related problems. This involves two key components: developing tools that are effective and acceptable to practitioners and establishing new policies at the management level to ensure that alcohol programs are mandated and adequately supported. Implementation science has demonstrated that successful adoption of new methods in healthcare requires support from research evidence ‘and’ strong leadership within the organization where implementation occurs [24]. While our study showed a modest increase in activity, it is unclear if this can be directly attributed to the new policy. Further research on combined implementation strategies will be essential in increasing alcohol-related activities effectively.

An important consideration based on the results of this study is that the policy change lacked structured information provided to primary care clinicians regarding the policy shift. Instead, it mainly involved a modification in the wording of the overall agreement governing publicly funded primary care. Our assessment indicates that such a policy shift, particularly without accompanying economic incentives or systematic monitoring of key indicators, is unlikely to result in significant changes in alcohol-related clinical activity. Additionally, it appears that only a small number of practitioners participated in the training sessions that were available. Attendance figures were not accurately recorded, as the training program was accessible online. Out of 1777 primary care professionals in Region Stockholm who completed voluntary course evaluations, only 21 were physicians. This attendance figure can be contrasted with the 1259 specialists in general medicine employed by the Region Stockholm [25].

The minimal impact of the new alcohol policy can be viewed from two distinct perspectives. Firstly, the lack of follow-up of the policy is evident. To our knowledge, the funding party (Region Stockholm) did not conduct systematic monitoring at the primary care clinic level regarding indicators examined in this study. Secondly, there was a deficiency in promoting the training courses effectively, as there was no clear leadership-driven campaign to endorse the courses. Despite the modest changes identified in this study, the introduction of the new alcohol treatment policy in Region Stockholm represents a promising initiative that aligns with fundamental principles of implementation science [11, 24]. Establishing a foundation for more effective policies is a commendable step, yet further efforts towards implementation are crucial for success.

### Strengths and limitations

The study has several strengths. It addresses key knowledge gaps in research on implementation of alcohol interventions. No previous study has investigated the effects of contractual requirements to include implementation of alcohol-related activities in primary care. This study is also novel as it investigated the potential predictive effects of both new requirements and the availability of web-based training for healthcare professionals in primary care. Another strength of the project is the use of registry data. Much research in implementation science, e.g. studies of the use of evidence-based guideline recommendations, relies on self-reported data on behaviours or intention to execute behaviours (e.g. adhering to a guideline). The use of registry data allows investigation into what healthcare professionals actually do rather than what they say they do [26]. The model developed for this study, with seven indicators on alcohol-related activities, can be used for further follow-ups in Region Stockholm as well as other jurisdictions.

The study also has shortcomings that must be considered. A weakness of the study is the lack of reliable information on how many of the professions from each primary care clinic participated in the training. Also, given the correlational nature of this study we cannot draw any conclusions regarding causality, i.e. whether the observed changes in the indicators were caused by policy change or the digital training courses—or by external factors.

## Conclusions

After the introduction of a new health care policy that included prevention and treatment of harmful use and dependence of alcohol in the agreement with the primary care clinics, these activities remained at low levels in primary care in Region Stockholm. The lack of enforcement and active support for the new policy likely played a role in this underwhelming result. A digital training program was introduced about 10 months after the new policy was introduced but it did not lead to a significant increase in alcohol-related clinical activity. Based on the results from this study, future efforts to identify and manage alcohol problems in primary care should provide clear incentives at the practitioner, local management, and stakeholder levels. Training should be prioritized at the clinic level. Regular follow-ups utilizing the indicators used in this study would provide management and clinical leadership with important feedback. This approach could help to further strengthen primary care as the basis of treatment for harmful use and dependence of alcohol.

## Supplementary Material

ckaf060_Supplementary_Data

## Data Availability

The data that support the findings of this study are available from the corresponding author upon reasonable request. Key points Prevention and treatment of alcohol dependence of moderate severity is feasible in primary care. A new policy mandating alcohol interventions in primary care and access to online training has not led to increased alcohol-related activity in primary care. The introduction of a new alcohol treatment policy is a promising initiative, yet further efforts towards implementation in clinical practice are crucial for success.
