# Supplementary material for: Addressing alcohol dependence in primary care: longitudinal registry-based study of practitioner activity following new policy and access to training
Source: Eur J Public Health. 2025 Jun 10;35(4):720–5. doi: 10.1093/eurpub/ckaf060 (PMC12311354; doi:10.1093/eurpub/ckaf060)
Supplement: ckaf060_Supplementary_Data [file ckaf060_supplementary_data.zip › ckaf060_Supplementary_Data/ejph-2024-09-om-0629-File007.docx]

Supplementary Figure 1. Trajectories for six alcohol-related indicators over measurement occasions
